# Supplementary material for: Association between oral microbiome and seven types of cancers in East Asian population: a two-sample Mendelian randomization analysis
Source: Front Mol Biosci. 2023 Nov 21;10:1327893. doi: 10.3389/fmolb.2023.1327893 (PMC10702768; doi:10.3389/fmolb.2023.1327893)
Supplement: Supplementary file 1 [file DataSheet1.ZIP › Supplementary Materials/MR plots of saliva/saliva═╝/ovarian cancer/pheno.429_to_ovarian cancer_leave_one_out.pdf]

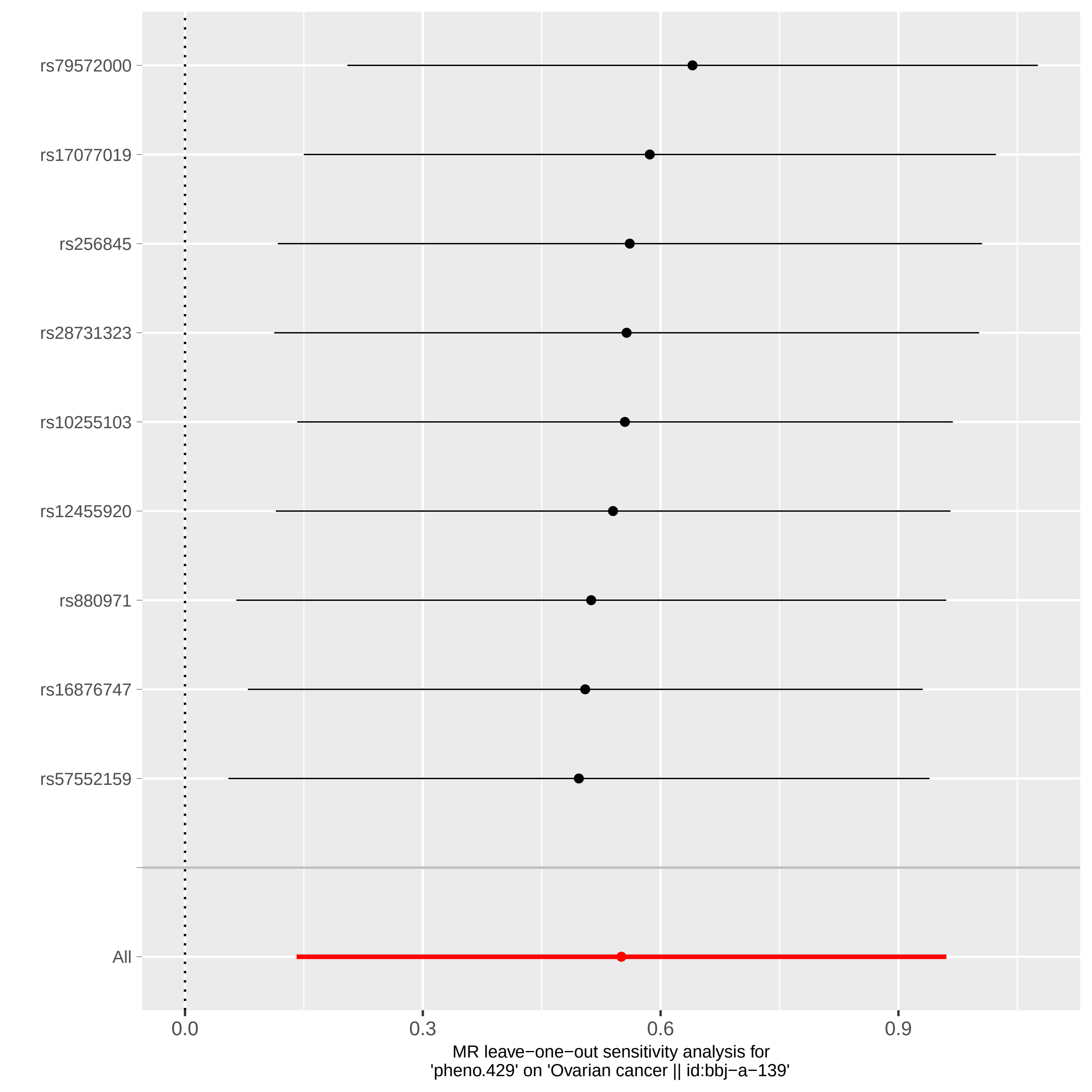
rs79572000
rs17077019
rs256845
rs28731323
rs10255103
rs12455920
rs880971
rs16876747
rs57552159
All
0.0
0.3
0.6
0.9
MR leave−one−out sensitivity analysis for
'pheno.429' on 'Ovarian cancer || id:bbj−a−139'
